# A2A: a platform for research in biomedical literature search

**DOI:** 10.1186/s12859-020-03894-8

**Published:** 2020-12-21

**Authors:** Maciej Rybinski, Sarvnaz Karimi, Vincent Nguyen, Cecile Paris

**Affiliations:** 1 CSIRO Data61, Sydney, Australia; 2 Australian National University, Canberra, Australia

**Keywords:** Clinical decision support, Evaluation, Information retrieval, Biomedical search, Genomics, Precision medicine, Software platform

## Abstract

**Background:**

Finding relevant literature is crucial for many biomedical research activities and in the practice of evidence-based medicine. Search engines such as PubMed provide a means to search and retrieve published literature, given a query. However, they are limited in how users can control the processing of queries and articles—or as we call them *documents*—by the search engine. To give this control to both biomedical researchers and computer scientists working in biomedical information retrieval, we introduce a public online tool for searching over biomedical literature. Our setup is guided by the NIST setup of the relevant TREC evaluation tasks in genomics, clinical decision support, and precision medicine.

**Results:**

To provide benchmark results for some of the most common biomedical information retrieval strategies, such as querying MeSH subject headings with a specific weight or querying over the title of the articles only, we present our evaluations on public datasets. Our experiments report well-known information retrieval metrics such as precision at a cutoff of ranked documents.

**Conclusions:**

We introduce the A2A search and *benchmarking* tool which is publicly available for the researchers who want to explore different search strategies over published biomedical literature. We outline several query formulation strategies and present their evaluations with known human judgements for a large pool of topics, from genomics to precision medicine.

## Background

The life sciences community is witnessing an unprecedented growth in the availability of textual resources. Citation repositories, such as PubMed [1], have recently been registering over a million new articles per year. That is, for PubMed, we see an increase of 2.4M records over a two year span, from early 2017 to early 2019. On the one hand, it means that there is more and more information at our fingertips. On the other hand, however, finding the relevant literature and scientific evidence in such volume of data is challenging.

The area of Computer Science which focuses on improving the mechanisms for searching for relevant information over a collection of resources, is known as Information Retrieval (IR). The access to information is crucial in life sciences; for example, scientists need to keep up with latest findings in science and contrast their work with reported findings, and medical professionals need to recommend adequate clinical trials to their patients. As a result, biomedical^1^ IR has become a prominent field of research. The need for better search systems has been widely recognised by both the IR and the life sciences communities [2].

Measuring the effectiveness of search systems requires thorough evaluations. One well-known body for setting common evaluation metrics and frameworks in the IR community is the Text Retrieval Conference (TREC) organised annually by the National Institute of Science and Technology (NIST). TREC has provided a number of biomedical and clinical evaluation tracks. These tracks include TREC Genomics (2003–2007) [3, 4], Medical track (2011–2012) [5], Clinical Decision Support (2014–2016) [6–10] (CDS) and its incarnation as TREC Precision Medicine (2017–2020) [11–13] (PM), as well as TREC-COVID [14].

While TREC has provided crucial resources, we believe that biomedical IR research is still hindered by three important factors: (1) the relatively low reproducibility of research methods and their reported results; (2) the software engineering component of the biomedical search; and, (3) the lack of universally accessible and reproducible baselines.

Undoubtedly, TREC shared tasks have led to a proliferation in IR research activity by introducing common grounds for evaluation. Nonetheless, the effectiveness of a search system comes from many complex design decisions, including: query formulation, document preprocessing, search engine implementation, and ranking methods. This leads to three evaluation obstacles. First, if we evaluate a complex search system, it is not possible to isolate the contribution of any specific technique or method without a dedicated ablation experiment.

Second, running IR experiments requires the deployment of a search engine, which in turn requires indexing documents, designing an index structure, parsing of source files, and relaying the search results to an evaluation tool. In other words, a biomedical natural language processing researcher, for example, with a prospectively interesting technique for query formulation such as Named Entity Detection with synonym expansion, has to set up an entire industry-grade infrastructure just to run a proof-of-concept experiment.

Finally, the third problem is that IR research involves taking many arbitrary decisions along the way, such as which engine to choose, or how to preprocess documents. Universally reproducible baselines are hardly attainable, and therefore new methods do not build upon existing research. This means that it is difficult to know if a new method improves retrieval effectiveness in general, or only when it is surrounded by components specific to a particular experiment.

We introduce an online tool, Apples-to-Apples^2^ (A2A), which alleviates those three problems. A2A provides an easy-to-use web-based Graphical User Interface (GUI), allowing users to define and execute biomedical IR experiments and explore their evaluation results.

A2A enables the evaluation of query formulation techniques within a predefined, yet parameterisable, framework. Evaluation results of each experiment can be easily reproduced by anyone by providing identical inputs to the system.

A2A’s GUI provides access to an extensible Python-based pipeline system which instantiates and evaluates IR systems with pre-indexed TREC collections, including a snapshot of the PubMed articles. This means users can establish baselines and evaluate query expansion strategies, while avoiding the tedious local deployment of all the components. Although A2A is principally suited for biomedical IR researchers, we believe that eliminating the technology obstacle is instrumental to making the field more accessible to a wider community of experts.

Finally, A2A provides predefined end-to-end parameterisable experiments, which leads to attainable and reproducible strong biomedical IR baselines. Additionally, the high adoption of our tool would mean crowd-sourcing the optimisation of those baselines to a broader community of researchers.

### Evaluation in IR

The lack of comparable results in IR has been observed and investigated in the IR community. A brief list of evaluation problems using TREC-like test collections can be found here [15]. Note that, here, we are not focusing on the test collection creation. While having widely accessible test collections is paramount in evaluating an IR solution, we cannot compare results obtained by different researchers without a unified platform and baseline benchmarks. We note that, in 2009, Armstrong et al. [16] investigated the problem of reporting improvements made over weak baselines in the *ad hoc* retrieval process tested in a TREC setting [17]. EvaluatIR, the platform Armstrong et al. proposed for comparing different IR systems,^3^ allowed researchers to upload the output of their systems and have them evaluated and compared against baselines. Unfortunately EvaluatIR is no longer publicly accessible. Another, more recent platform, is provided by EvALL [18]. In this system, some of the existing shared tasks are benchmarked, and new benchmark data can be uploaded, based on local executions. Inspired by these systems, we created a platform that allows the testing of a variety of retrieval methods on the TREC corpora. While EvALL is a generalised platform focused on benchmarking locally deployed systems (so, it provides a web-based evaluation module), we focus solely on biomedical IR, with its unique challenges and methods, which include dealing with biomedical vocabularies such as Unified Medical Language System (UMLS) and Medical Subject Headings (MeSH). We also provide a mechanism for the remote execution of end-to-end baseline experiments.

### Open source and public retrieval engines

There are a number of open source search engines available to the IR community, such as Apache Solr,^4^ Lucene,^5^ Elastic search^6^, Terrier [19], and Galago [20], which can be used by professional developers and researchers with information retrieval background. However, there is a steep learning process in setting these up for even a basic retrieval task.

Closest to our work is a toolkit called Anserini [21], built on Lucene. It provides wrappers for IR researchers to build web applications and run experiments using the standard TREC collections. While an invaluable resource, it still requires development knowledge some researchers might not have. Our vision is similar, as we are concerned about the reproducibility of reported methods, especially in the biomedical domain. However, we also intend to make it possible for anyone with interest in biomedical search to be able to run experiments without setting up a search engine. We introduced A2A system recently as demonstrations at the SIGIR^7^ conferences [22, 23]. Here, we provide a thorough description of the system together with some substantial changes in what is on offer for benchmarking.

## Implementation of the A2A system

A2A is a web application. It allows for the execution of experiments on various life sciences themed IR benchmark collections facilitated by NIST within the TREC framework. The system’s back-end implements an IR pipeline which, given an input of TREC formatted topics, follows these steps: Query processing, search, filtering (optional), and evaluation.

**Fig. 1:**
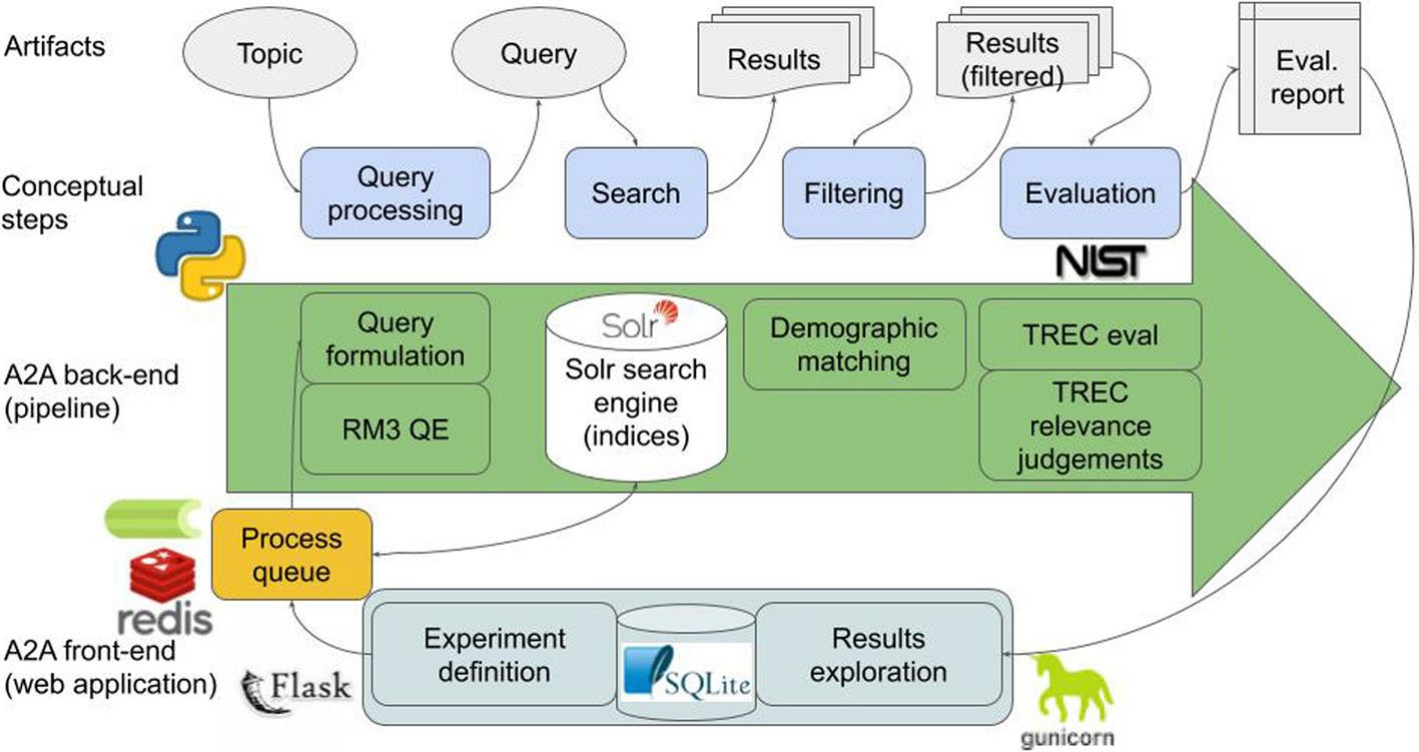
An overview of the A2A benchmarking system. We present a layered view with key interactions between the elements. Nore that the role of the user in an IR benchmarking scenario is to define the experiment (a combination of topics, document collection, human judgements, and a retrieval model with its parameters), which is accomplished through A2A front-end (bottom layer). The processing path leading from a topic to the evaluation report is presented in the *Artifacts* an *Conceptual steps* layers; the former layer presents the intermediate entities (artifacts) obtained at respective steps of the latter layer. The *A2A back-end layer* lists physical elements of the system’s pipeline involved at respective conceptual steps. The front end application starts an instance of the A2A back-end pipeline, parameterised per user request; a process queue is used for asynchronous scheduling and execution of the pipeline process. The back-end pipeline is implemented in Python 3.7, with Apache Solr search engine. We use NIST-supplied TRECeval script to calculate metrics. The process queue is implemented with Celery and Redis. The front-end application is implemented in Flask and deployed on a gunicorn web server

The overview of the system’s architecture and the relationships between individual modules and the processing steps is presented in Fig. 1.

**Fig. 2:**
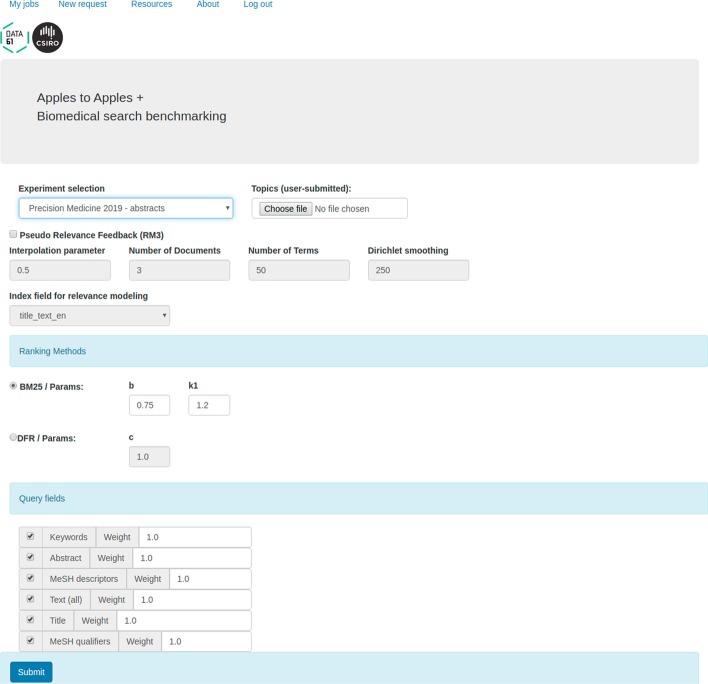
An A2A screen for setting parameters of a new experimental run

**Fig. 3:**
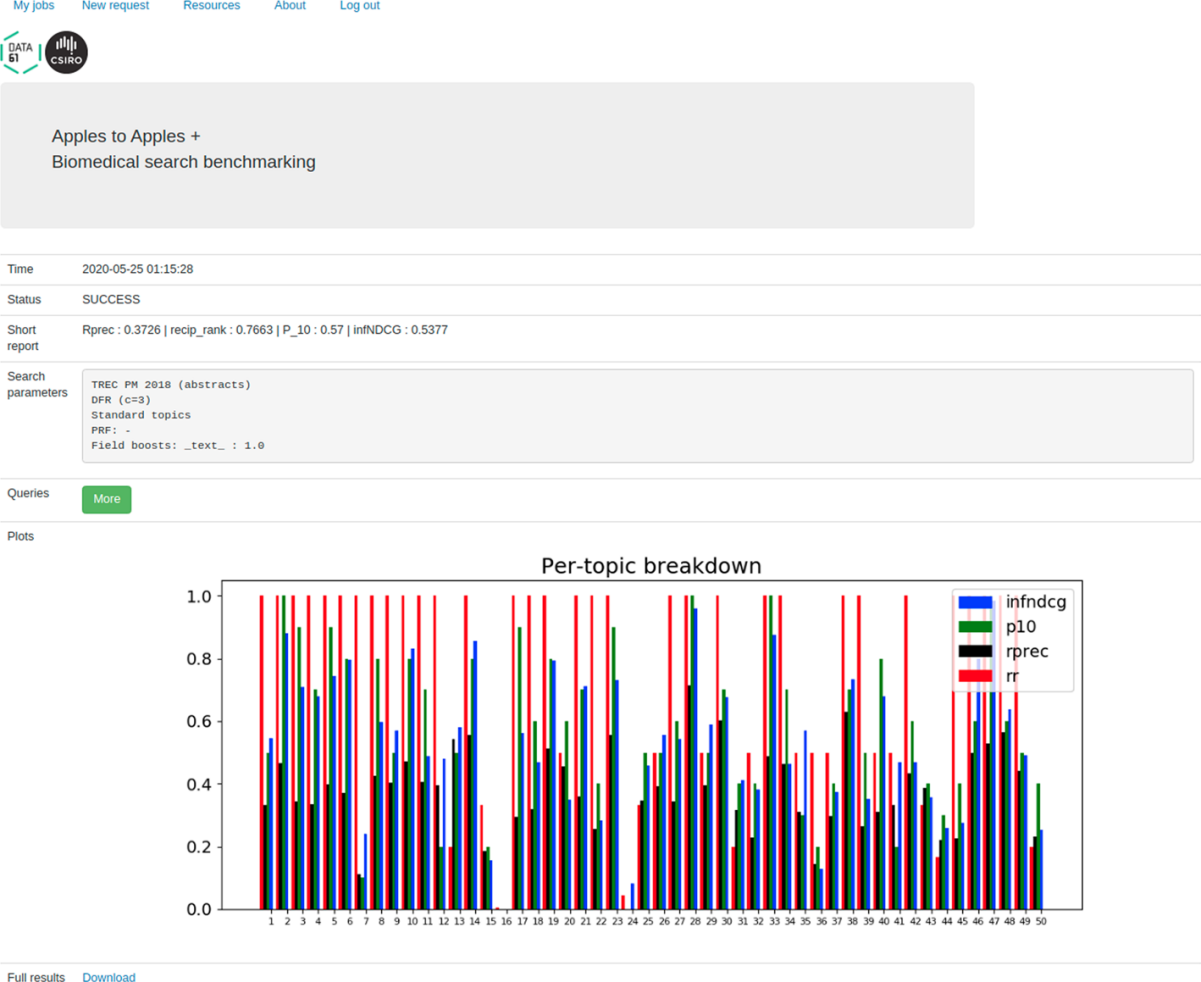
An A2A screen for visualisation of results of an experiment (a ‘single job view’)

The front-end enables users to parameterise and execute their experimental runs and then examine the results. The system allows users to request experimental runs which employ one of the predefined query formulation strategies or to skip the automatic query formulation step altogether by submitting user-defined queries generated offline. The screen for registering a new experimental run is presented in Fig. 2; the interface to examine the corresponding results is presented in Fig. 3. The interface for browsing through the individual documents which contribute to the results is presented in the “Discussions” section (Use Case 5).

Each of the following parts of this section details a specific aspect of the system and its significance to the individual pipeline steps. We also briefly present materials and methods pertinent to each of these system components. Finally, each of the subsections includes key implementation and software architecture notes.

### Datasets and indexing

All the TREC collections used in A2A consist of a set of topics and at least one corpus of documents with a corresponding set of human-produced relevance judgements for topic-document pairs. Each of the corpora is indexed in the Solr 8.2.0 search engine using its standard document preprocessing mechanisms (stopword elimination, stemming and case normalisation). As A2A is focused solely on benchmarking search systems for life sciences, we considered only TREC tasks representing *ad hoc* document retrieval problems. A summary of the TREC tasks featured in A2A together with corresponding indexed corpora (there is a many-to-many relationship) is presented in Table 1. Table 2 presents an example topic from each of the TREC collections.

**Table 1:** Overview of tasks and corpora included in A2A

Corpus	Fields	# docs	Task
2004 PubMed snapshot	*title, abstract, chemicals, MeSH terms*	4.59M	**Genomics 2004**
			50 topics, 8268 judgments, literature retrieval with biology topics
			**Genomics 2005**
			50 topics, 39958 judgments, literature retrieval with biology topics
2014 PMC snapshot	*full-text, abstract, keywords, title, MeSH descriptors, MeSH qualifiers, MetaMap (title), MetaMap (abstract)*	0.73M	**CDS 2014 (Description)**
			30 topics, 37949 judgments, literature retrieval with clinical case descriptions
			**CDS 2014 (Summary)**
			30 topics, 37949 judgments, literature retrieval with clinical case summaries
			**CDS 2015 (Description)**
			30 topics, 37807 judgments, literature retrieval with clinical case descriptions
			**CDS 2015 (Summary)**
			30 topics, 37807 judgments, literature retrieval with clinical case summaries
2016 PMC snapshot	*full-text, abstract, keywords, title, MeSH descriptors, MeSH qualifiers, MetaMap (title), MetaMap (abstract)*	1.25M	**CDS 2016 (Description)**
			30 topics, 37707 judgments, literature retrieval with clinical case descriptions
			**CDS 2016 (Summary)**
			30 topics, 37707 judgments, literature retrieval with clinical case summaries
			**CDS 2016 (Note)**
			30 topics, 37707 judgments, literature retrieval with clinical notes
2017 ClinicalTrials.gov	*brief summary, brief title, clinical trial ID, detailed description, drug name, drug keywords, exclusion, gender, general keywords, inclusion, intervention type, maximum age, minimum age, official title, and primary outcome*	0.24M	**PM 2017 (clinical trials)**
			30 topics, 13019 judgments, clinical trial retrieval with patient profiles
			**PM 2018 (clinical trials)**
			50 topics, 14188 judgments, clinical trial retrieval with patient profiles
2019 ClinicalTrials.gov	*brief summary, brief title, clinical trial ID, detailed description, drug name, drug keywords, exclusion, gender, general keywords, inclusion, intervention type, maximum age, minimum age, official title, and primary outcome*	0.3M	**PM 2019 (clinical trials)**
			40 topics, 12996 judgments, clinical trial retrieval with patient profiles
2017 abstracts	*title, abstract, keywords, article type, MeSH descriptors, MeSH qualifiers*	26.73M	**PM 2017 (abstracts)**
			30 topics, 22642 judgments, literature retrieval with patient profiles
			**PM 2018 (abstracts)**
			50 topics, 22429 judgments, literature retrieval with patient profiles
2019 PubMed snapshot	*title, abstract, keywords, article type, MeSH descriptors, MeSH qualifiers*	29.13M	**PM 2019 (abstracts)**
			40 topics, 18316 judgments, literature retrieval with patient profiles

**Table 2:** Topic examples for different TREC tasks included in A2A

Row	Dataset	Topic example
1	Genomics 2004	**Title**: Ferroportin-1 in humans. **Need**: Find articles about Ferroportin-1, an iron transporter, in humans. **Context**: Ferroportin1 (also known as SLC40A1; Ferroportin 1; FPN1; HFE4; IREG1; Iron regulated gene 1; Iron-regulated transporter 1; MTP1; SLC11A3; and Solute carrier family 11 (proton-coupled divalent metal ion transporters), member 3) may play a role in iron transport.
2	Genomics 2005	**Narrative**: Describe the procedure or methods for how to “open up” a cell through a process called “electroporation.”
3	CDS 2014	**Type**: diagnosis. **Description**: A 58-year-old nonsmoker white female with mild exertional dyspnea and occasional cough is found to have a left lung mass on chest x-ray. She is otherwise asymptomatic. A neurologic examination is unremarkable, but a CT scan of the head shows a solitary mass in the right frontal lobe. **Summary**: 58-year-old female non-smoker with left lung mass on x-ray. Head CT shows a solitary right frontal lobe mass.
4	CDS 2015	**Type**: test. **Description**: A 32 year old female with no previous medical history presents to clinic to discuss lab results from her most recent pap smear. She reports no complaints and is in general good health. The results of her PAP were cytology negative, HPV positive. **Summary**: A 32 year old female with screening that was positive for human papilloma virus with normal Pap smears.
5	CDS 2016	**Type**: diagnosis. **Note**: 78 M w/ pmh of CABG in early [**Month (only) 3**] at [**Hospital6 4406**] (transferred to nursing home for rehab on [**12-8**] after several falls out of bed.) He was then readmitted to [**Hospital6 1749**] on [**3120-12-11**] after developing acute pulmonary edema/CHF/unresponsiveness?. There was a question whether he had a small MI; he reportedly had a small NQWMI. He improved with diuresis and was not intubated. Yesterday, he was noted to have a melanotic stool earlier this evening and then approximately 9 loose BM w/ some melena and some frank blood just prior to transfer, unclear quantity. **Description**: 78 M transferred to nursing home for rehab after CABG. Reportedly readmitted with a small NQWMI. Yesterday, he was noted to have a melanotic stool and then today he had approximately 9 loose BM w/ some melena and some frank blood just prior to transfer, unclear quantity. **Summary**: A 78 year old male presents with frequent stools and melena.
6	PM 2017	**Disease**: Liposarcoma. **Gene**: CDK4 Amplification. **Demographic**: 38-year-old male . **Other**: GERD
7	PM 2018	**Disease**: melanoma. **Gene**: BRAF (V600E). **Demographic**: 64-year-old male
8	PM 2019	**Disease**: prostate cancer. **Gene**: ATM deletion. **Demographic**: 50-year-old male

The corpus of the TREC Genomics 2004 *ad hoc* retrieval task is a large subset of the PubMed citation database records—the corpus includes over 4.5M records marked as complete between 1994-2003 (inclusive). The 50 topics represent real information needs reported by biology and biomedical researchers. Each topic consists of the *title*, *need*, and *context* fields, all expressed in natural language. An example topic is included in row 1 of Table 2.

The documents of the TREC Genomics 2004 corpus are indexed with the following fields: *title*, *abstract*, *chemicals*, and *MeSH terms*. All the information, including lists of chemical compounds mentioned in the text, were supplied with the corpus.

The TREC Genomics 2005 *ad hoc* retrieval task uses the same corpus of documents as the 2004 edition of the track, with a set of 50 new topics. Each of the 2005 topics contains a single textual field. Although the 2005 topics also represent real information needs reported by biologists, they were created artificially by inserting texts corresponding to biomedical entities and phenomena into predefined query templates. An example can be found in row 2 of Table 2.

TREC CDS 2014 includes a corpus of 733,138 full-text articles in the January 21 2014 snapshot of the PubMed Central repository. The collection includes 30 topics created by National Library of Medicine (NLM) experts to serve as idealised representations of medical cases. Each topic consists of the information need type (diagnosis, treatment advice, tests advice) to represent different scenarios prevalent in clinical decision support. Additionally, each of the topics contains two fields: *description* and *summary*. The former includes a full account of the medical report represented by the topic, and the latter is a brief summarisation of key facts. Since TREC guidelines for CDS 2014 postulate using only one of the fields for all topics within a single evaluation run, there are *de facto* two separate retrieval tasks with one for descriptions, and one for summaries. For an example of a CDS 2014 topic, the reader may refer to the third row of Table 2.

TREC CDS 2014 documents are indexed with the following fields parsed directly from the corpus files: *full-text*, *abstract*, *keywords* and *title*. Additionally, MeSH terms were retrieved by cross-querying PubMed and indexed as *MeSH descriptors* and *MeSH qualifiers*. Finally, UMLS concepts were ‘mined’ from abstracts and titles at indexing time (using the MetamapLite tool^8^) and appended to the index as *Metamap of title* and *Metamap of abstract*.

TREC CDS 2015 reuses the corpus of TREC CDS 2014, while providing a set of 30 new topics. The 2015 topics are structured in the same way as the 2014 topics and identical evaluation guidelines apply. Additionally, TREC CDS 2015 introduces task ‘B’, in which the patient’s diagnosis is known for those topics that seek testing or treatment advice. Although task ’B’ is not explicitly reflected in the A2A system, the “Discussions” section provides a walk-through on how to run such an experiment using the official TREC task B topics and minimal offline processing.

The corpus of TREC CDS 2016 is a snapshot of literature from PubMed Central taken on 28 March 2016. It contains 1.25 million full-text journal articles. For CDS 2016, 30 new topics are based on actual admission notes (extracted from HPI—History of Present Illness—fields from MIMIC-III database [24]). Each topic has three different fields: (1) *note*, or the original clinical note; (2) *description*, a simplified version of note, where all abbreviations and jargon are removed (so, similar to the *description* field from previous editions of CDS); and, (3) *summary*, a condensed version of the description. Evaluation guidelines for CDS 2016 also advise the use of only one field type per single evaluation. See row 5 of Table 2 for an example topic.

The indexing procedure and document composition for TREC CDS 2016 is identical to that of 2014, so the index structure (schema) is the same.

TREC PM 2017 introduces two tasks, each with its own document corpus. In the literature abstract retrieval task, the corpus consists of over 26.5M PubMed abstracts (a January 2017 snapshot), supplemented with over 60K abstracts from top oncology conferences (AACR and ASCO). The abstract retrieval task is focused on searching for treatment information. The second task of TREC PM 2017 is a clinical trial retrieval task, focused on assigning prospective/relevant clinical trials to oncology patients (with each topic representing a patient). The corpus of clinical trials consists of over 241K real clinical trials’ records (an April 2017 snapshot of ClinicalTrials.gov database). Both tasks of TREC PM 2017 share the same set of 30 topics prepared by precision oncologists from the University of Texas and the Oregon Health and Science University. Each topic represents an individual oncology patient. The topics of PM 2017 contain four fields: *disease* (i.e., a cancer type), *gene* (a patient-specific genetic mutation or feature), *demographic* (age and gender information), and *other* (other possibly relevant information, such as comorbidities). In the TREC PM evaluations, all fields can be used to formulate a query.

For TREC PM 2017, literature abstracts are indexed with *title* and *abstract* fields. Additionally the abstracts from PubMed are indexed with corresponding *keywords*, *article types*, *MeSH descriptors*, and *MeSH qualifiers*, all extracted directly from the source files of the corpora.

We index clinical trials with the following fields: *brief summary*, *brief title*, *clinical trial ID*, *detailed description*, *drug name*, *drug keywords*, *exclusion*, *gender*, *general keywords*, *inclusion*, *intervention type*, *maximum age*, *minimum age*, *official title*, and *primary outcome*. Age-related fields are numeric, and all other fields except *ID* are copied into an aggregate *text* field, which is also indexed. Inclusion and exclusion criteria are parsed from a single field in original documents using regular expressions. The document structure and indexing procedure are identical for the TREC PM 2017 and the 2019 clinical trials collections.

TREC PM 2018 uses the same corpora as TREC PM 2017 in both (abstract and clinical trials) retrieval tasks. The collection contains 50 new synthetic topics, also created by precision oncologists. The topics are structured like those of TREC PM 2017, but without the *other* field.

TREC PM 2019 uses updated versions of corpora used in 2017 and 2018 editions of TREC PM. The literature abstracts corpus is a December 2018 snapshot of PubMed (over 29M abstracts), while the clinical trials corpus is a May 2019 snapshot of ClinicalTrials.gov database (over 305K clinical trials). The 40 new topics of the track are structured identically to those of 2018—the novelty of the 2019 edition being that the last 10 topics are focused on genetically-factored disorders other than cancer.

The abstracts of TREC PM 2019 are all PubMed records, so our index procedure mirrors that of the TREC PM 2017 PubMed abstracts.

### Query processing methods

By ‘query processing’, we mean all the processing steps that transform the contents of a TREC topic into a query text, which is then submitted to the actual search engine (Solr). One of the key features of the A2A system is the possibility of running experiments with offline query processing to evaluate its impact on retrieval performance. In this subsection, however, we only present the automated query processing techniques, which serve as baseline techniques of query formulation. Some alternatives, i.e., examples of offline query formulation strategies are presented in the "Discussions" section to illustrate the experimental capabilities of A2A.

#### Automated baselines

We propose simple default query formulation strategies for each of the evaluation tasks available in the system. The overview of these strategies is presented in Table 3, with examples built from topics of Table 2.

**Table 3:** Summary of predefined query formulation strategies for each of the tasks in A2A with examples

Row	Task	Composition	Query
1	Genomics 2004	Title $+$ Need $+$ Context	(Ferroportin-1 in humans) (Find articles about Ferroportin-1, an iron transporter, in humans.) (Ferroportin1 (also known as SLC40A1; Ferroportin 1; FPN1; HFE4; IREG1; Iron regulated gene 1; Iron-regulated transporter 1; MTP1; SLC11A3; and Solute carrier family 11 (proton-coupled divalent metal ion transporters), member 3) may play a role in iron transport)
2	Genomics 2005	Narrative	Describe the procedure or methods for how to “open up” a cell through a process called “electroporation”
3	CDS 2014 (Description)	Description	A 58-year-old nonsmoker white female with mild exertional dyspnea and occasional cough is found to have a left lung mass on chest x-ray. She is otherwise asymptomatic. A neurologic examination is unremarkable, but a CT scan of the head shows a solitary mass in the right frontal lobe
4	CDS 2014 (Summary)	Summary	58-year-old female non-smoker with left lung mass on x-ray. Head CT shows a solitary right frontal lobe mass.
5	CDS 2015 (Description)	Description	A 32 year old female with no previous medical history presents to clinic to discuss lab results from her most recent pap smear. She reports no complaints and is in general good health. The results of her PAP were cytology negative, HPV positive
6	CDS 2015 (Summary)	Summary	A 32 year old female with screening that was positive for human papilloma virus with normal Pap smears
7	CDS 2016 (Note)	Note	78 M w/ pmh of CABG in early [**Month (only) 3**] at [**Hospital6 4406**] (transferred to nursing home for rehab on [**12-8**] after several falls out of bed.) He was then readmitted to [**Hospital6 1749**] on [**3120-12-11**] after developing acute pulmonary edema/CHF/unresponsiveness?. There was a question whether he had a small MI; he reportedly had a small NQWMI. He improved with diuresis and was not intubated. Yesterday, he was noted to have a melanotic stool earlier this evening and then approximately 9 loose BM w/ some melena and some frank blood just prior to transfer, unclear quantity
8	CDS 2016 (Description)	Description	78 M transferred to nursing home for rehab after CABG. Reportedly readmitted with a small NQWMI. Yesterday, he was noted to have a melanotic stool and then today he had approximately 9 loose BM w/ some melena and some frank blood just prior to transfer, unclear quantity
9	CDS 2016 (Summary)	Summary	A 78 year old male presents with frequent stools and melena
10	PM 2017	Disease $+$ Gene	Liposarcoma CDK4 Amplification
11	PM 2018	Disease $+$ Gene	melanoma BRAF (V600E)
12	PM 2019	Disease $+$ Gene	prostate cancer ATM deletion

Those basic strategies can be optionally complemented with a Pseudo-Relevance Feedback (PRF) based query expansion method known as RM3 [25]. RM3 is a well-known and proven statistical method for query expansion/reformulation, and therefore constitutes an important baseline for measuring query reformulation performance.

The intuition behind RM3 is that it uses the original query (so, in our case, one created using one of our basic strategies) and retrieves the top *k* documents from the search engine according to some ranking function *r* (see next subsection for more information on ranking functions). It then uses the terms (words) found in those top *k* documents and scores assigned to these documents by a ranking function *r* to create a term-weight assignment—a relevance model. This relevance model, truncated at *m* highest-scored terms, is then interpolated with importance weights (e.g., uniform) of the original query to create the final (expanded) representation of the query.

More formally, given a query represented with a bag^9^ of terms *Q*, RM3 can be defined as assigning probabilities *P*(*t*|*Q*) to each term *t* of $D_R \bigcup Q$, where $D_R$ are the top *k* documents retrieved for query Q, according to the ranking function *r*. *P*(*t*|*Q*) is

1 $$\begin{aligned} P(t|Q) = (1-\alpha ) \cdot \frac{\sum _{D \in D_R} P(t|D) \cdot r(D,Q)}{\sum _{t \in D_R \bigcup Q}(\sum _{D \in D_R} P(t|D) \cdot r(D,Q))} + \alpha \cdot P_0(t|Q), \end{aligned}$$

where T is the set of all terms of $D_R$, and *r*(*D*, *Q*) is the relevance score of *D* given *Q* according to the underlying search engine’s ranking function *r*; $\alpha$ is a free interpolation parameter from interval [0, 1]. $P_0(t|Q)=\frac{f(t,Q)}{|Q|}$, where *f*(*t*, *Q*) denotes frequency of term *t* in *Q*. Additionally, *P*(*t*|*D*) is

2 $$\begin{aligned} P(t|D)=\frac{f(t,D) + \mu \frac{f(t, D_R)}{|D_R|}}{|D| + \mu }, \end{aligned}$$

where *f*(*t*, *D*) denotes frequency of term *t* in document *D*, $f(t,D_R)$ is frequency of term *t* in $D_R$ and |*D*|, and $|D_R|$ denote word-lengths; $\mu$ is another free parameter (Dirichlet smoothing).

In A2A, the end-user also chooses which index field should be used to represent documents from $D_R$ (e.g., *abstract* or *title*). For example, if we take the query from last row, last column of Table 3 (‘prostate cancer ATM deletion’), RM3 with parameters $\alpha =0.3, k=3, m = 8, \mu =100$ and *abstract* field used for relevance modeling can produce a final query representation like the one below:

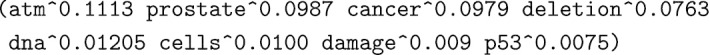



The topic of document ranking functions is addressed later in this section. For the sake of completeness, we note that we use the same ranking function r (with the same free parameters) within RM3 and in the final search with the expanded query. We sacrifice the possibility of fully parameterising the RM3 runs for the overall simplicity of the system.

### Document ranking functions

A2A’s experimental setup offers a choice between two document ranking functions: Divergence from Randomness (DFR) [26] and Okapi Best Match 25 (BM25) [27]. This choice dictates which mechanism is used to calculate the document relevance. That is, it determines the way the final document ranking is produced by Solr search engine.

Inclusion of BM25 is due to the method’s status as a *de facto* standard in IR. BM25 constitutes a strong baseline, which often outperforms elaborate ranking strategies [28]. Our choice of the second method, DFR, has been influenced by its high effectiveness reported for biomedical search [29, 30].

BM25 is a refined variant of Term Frequency-Inverse Document Frequency (TF-IDF). The core idea of BM25 is that documents should be assigned higher scores the more important terms (words) of the query they contain, where the importance of an individual term is modeled by its inverse document frequency (so, an inverse of how common it is in the entire corpus). Formally, given a query *Q* (again, a bag) with terms $t_1, t_2, ..., t_n$, the BM25 score of a document *D* is given by

3 $$\begin{aligned} BM25(D, Q) = \sum _{i=1}^{n} IDF (t_i) \cdot \frac{f(t_i, D) \cdot (k_1 + 1)}{f(t_i, D) + k_1 \cdot (1 - b + b \cdot \frac{|D|}{avgdl})}, \end{aligned}$$

where $f(t_i, D)$ denotes the raw frequency of term $t_i$ in D, $k_1$, and *b* are free parameters set by the user via A2A interface, |*D*|, is the length of *D*, and *avgdl* is an average document length for the corpus; the inverse document frequency of $t_i$, $IDF(t_i)$ is

4 $$\begin{aligned} IDF(t_i)=\frac{N - n_{t_i} + 0.5}{n_{t_i} + 0.5}, \end{aligned}$$

where N is the total number of documents in the corpus, and $n_{t_i}$ is the number of documents which contain $t_i$.

The divergence from randomness is a family of probabilistic models based on the idea that term informativeness (another proxy for importance) can be quantified as the divergence of the term’s distribution from a random distribution. The model we use in A2A is commonly referred to as InL2 (Inverse Document Frequency model with Laplace after-effect and Normalization 2). Since it is the only DFR model used in A2A, we refer to it simply as ‘DFR’. Building on the already introduced notation, the DFR score of a document *D*, given a query *Q* (with terms $t_1, t_2, ..., t_n$ ) is

5 $$\begin{aligned} DFR(D,Q)= \sum _{i=1}^{n} 1/|Supp(Q)| \frac{1}{f'(t_i,D)+1}\left( f'(t_i,D) \cdot log \frac{N+1}{n_{t_i}+0.5}\right) , \end{aligned}$$

where |*Supp*(*Q*)| denotes the number of distinct terms in *Q*, and $f'(t_i, D)$ is given by

6 $$\begin{aligned} f'(t_i, D)=f(t_i, D) \cdot log\left( 1 + c \cdot \frac{avgdl}{|D|}\right) , \end{aligned}$$

where $c>0$ is a hyperparameter set by the user via the A2A interface.

The scoring functions presented above assume single-fielded document representations. In A2A we use multi-fielded indices with Solr’s implementation of edismax parser,^10^ which means that the final document score is computed as a maximum of scores calculated for the individual indexed fields. In combination with the option of index field boosting, document scores are calculated as a maximum of user-weighted field scores.

This can be illustrated with the following simple example. In an index with two fields, e.g., *abstract* and *title*, if the user chooses to use DFR with abstract weight $w_a$ and title weight $w_t$, the final score *s*(*Q*, *D*) will be calculated as:

7 $$\begin{aligned} s(Q, D) = max ( w_a \cdot DFR_a(D_a,Q), w_t \cdot DFR_t(D_t, Q) ), \end{aligned}$$

where $D_a$ is an abstract of document *D*, and $D_t$ is its title; $DFR_a$ denotes the DFR score calculated in the space of abstracts (in particular normalised with lengths corresponding to abstracts in the corpus and the number of abstracts in the corpus), and $DFR_t$ denotes the DFR score calculated in the space of titles. This illustrative example can be easily generalised to other scoring formulae (i.e., BM25) and different sets of fields present in an index (for example, *title*, *abstract*, *chemicals*, *MeSH terms*, as in the index for TREC Genomics).

A2A, through a user-specified parameter, allows for strict boolean matching of demographic criteria from the TREC PM tasks’ topics to structured information present in clinical trials corpus. It results in filtering out those documents (trials) that do not match the patient’s age or gender.

### Evaluation metrics

To evaluate experimental runs against human-produced relevance judgements, A2A runs the NIST-provided tools in the background (trec-eval, sample-eval), returning some of the evaluation metrics commonly used in TREC evaluations.

Our evaluations follow those metrics suggested by the TREC organisers. For all experiments we calculate precision at 10 (P@10), recall-precision (R-Prec) and reciprocal rank (RR). For experiments which support sampled measures, A2A also returns inferred normalised discounted cumulative gain (infNDCG) [31]. A2A presents both results averaged over all the topics in the testing sets, as well as a topic-by-topic breakdown of the evaluation results.

P@10 is defined as the proportion of relevant (non-zero relevance judgment) documents in the top 10 documents of the ranking returned for a given topic. R-Prec is the proportion of relevant documents at *rel*, where *rel* is the total number of relevant documents for the topic. RR is defined as the inverse of the ranking position of the highest ranked true positive (so: $RR=1$, if the first truly relevant document in ranking is at the top position; $RR=0.5$ if the top ranked truly relevant document comes second, etc.). The calculation method of infNDCG metric is presented in detail by Yilmaz et al. [31].

### Web application and software architecture

A2A is developed as a Python-based web application with mostly python-based back-end. From a deployment and software architecture standpoint, the system has 4 main components:

- A Flask web application running on gunicorn server;
- An SQL database, which registers users and their requests;
- A Solr 8.2.0 server, providing offline indexing and online search;
- A Celery job queue, which instantiates the IR pipeline process (implemented in Python); the pipeline’s main function is to oversee communications with Solr, per user requests.

The Celery job queue acts as a link between the user interface and the actual search logic implemented through a Python-based pipeline system.

The SQL database has minimal structure—a ‘users’ table and ‘jobs’ table, connected with a 1:m relationship. The ‘users’ table handles login credentials and provides the master key to determine which requests are run/owned by whom. The ‘jobs’ table records successful and unsuccessful executions requested by the users, as well as the parameters of these requests. It also points to the results of successfully executed jobs, as these are stored in the A2A server’s filesystem.

The web application provides a navigation bar at the top of every page, linking to the following pages:

- ‘My jobs’ (login required)—a screen providing a table view of all jobs requested by the user with execution status and abbreviated results report for the successfully completed ones; it provides buttons to either remove or switch to the detailed view of a specific job;
  - Single job view (login required; accessed through ‘My jobs’)—apart from the information already available in the table view, the page reports a full list of input parameters, topic-by-topic evaluation results and final version of queries submitted to the Solr search engine; it also provides a link to download the TREC-formatted search and evaluation result files and a link redirecting to a visual exploration of the search results (‘results view’).
  - Results view (login required; accessed through single job view)—a screen allowing the user to explore top 50 (per topic) results of a given successfully completed request; the interactive table was implemented using DataTables jQuery plugin^11^
- ‘New request’ (login required)—a screen providing an interface for specifying the parameters of a new experimental run and requesting its execution;
- ‘Resources’ (login required)—a page with download links to additional resources: TREC topic files, example scripts for topic processing and query formulation (discussed in more detail in the next section) and some examples of the results (modified topic files) produced by these scripts;
- ‘About’—a page with basic information about the system; and,
- ‘Log out’.

Accessing the login protected content without being logged in triggers a redirection to the login/new-user-registration page.

To run an experiment with a user-modified topic file, the ‘Topics (user-submitted)’ dialog has to be used to choose the file from a local computer. Topics submitted by users can contain any number of topics fields, but are expected to contain exactly one field of type *user_query*. The contents of this field are relayed directly to the Solr search engine via the edismax parser. As a result, A2A users can run elaborate query reformulation experiments relying only on simple XML parsing and basic text processing techniques. Some examples are presented in the following section.

## Discussions

In this section, we provide a detailed description of several use cases to present the capabilities of the A2A system. The in-built processing of A2A provides an interface to run a variety of in-built baseline experiments on biomedically themed TREC collections. While this core functionality is important, the system is easy to use and we believe that the information from the previous section is sufficient to set up basic experiments. Here, we focus mostly on more elaborate scenarios, with a guide through for example experiments involving offline topic processing. Use cases 1–4 show how different retrieval strategies can be evaluated using the proposed system. The techniques include topic/index field boosting (use case 1), rule-based query expansion (use case 2), automated query expansion using external knowledge (use cases 3 and 4). Each of the use cases outlines the set-up of the experiment and presents the evaluation results as obtained with the A2A system. Use case 5 outlines an application of our system to ‘free’ search (with an arbitrarily chosen query formatted as an XML input) and illustrates the A2A’s capacity of document-level exploration of the results.

The second part of this section provides a detailed discussion of the advantages of A2A over other related tools. We also discuss some of the current limitations of the system.

### Use cases

#### Use case 1: topic and index field boosting

One of the techniques reported commonly by participants in the TREC shared tasks is the selection of boosts/weights specific to a particular topic and index fields. Index field boosting is supported in the predefined A2A pipeline (as weights for individual index fields). Topic field boosting is not reflected explicitly in the user interface, but it can be easily implemented with user-modified topic files and query boosting syntax supported by the edismax parser.

In the mini-experiment presented here, we address the TREC PM 2018 abstract retrieval task. We perform a coarse search over parameters to find a strong DFR system ($c=3$, $w_{{text}}=1$, weights 0 for other fields; we refer to it as ’DFR-strong’ for the remainder of this use case) that improves over the baseline ($c=1$, weights 1 for all fields) in three out of four evaluation metrics. We want to establish if boosting a specific topic field (*gene* or *disease*) produces additional improvement. Pseudocode for the topic processing is presented below (a Python implementation of the script is included in the Resources page—topic-fields-reweighting.py):

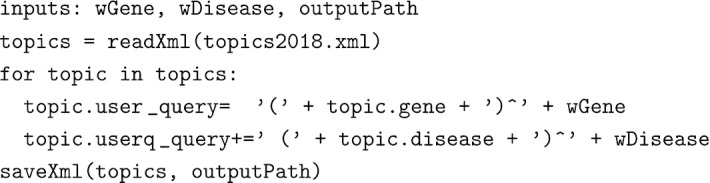



We evaluate topic field boosting with DFR-strong settings for weights W in (0, 0.1, 0.2, 0.3, 0.4, 0.6, 0.7, 0.8, 1), where $wGene=W$ and $wDisease=1-W$. The respective modified topic files can also be found in the Resources page of A2A.

**Table 4:** Results for use case 1: search effectiveness for different weights (boosts) for *gene* (*W*) and *disease* ($1-W$) fields for TREC 2018 collection (abstract retrieval), compared with a strong DFR baseline (DFR-strong)

Run	RPrec	RR	P@10	InfNDCG
DFR-strong	0.373	0.766	0.570	0.538
$W=0$	0.009	0.049	0.012	0.012
$W=0.1$	0.264	0.728	0.516	0.424
$W=0.2$	0.327	0.745	0.550	0.494
$W=0.3$	0.356	0.719	0.578	0.529
$W=0.4$	0.367	0.736	0.562	0.534
$W=0.6$	0.359	0.725	0.566	0.508
$W=0.7$	0.341	0.710	0.546	0.484
$W=0.8$	0.312	0.722	0.508	0.451
$W=0.9$	0.284	0.634	0.452	0.409
$W=1$	0.101	0.328	0.214	0.180

The results of the experiment are presented in Table 4. This simple evaluation indicates that topic field re-weighting does not improve search performance with the DFR-strong baseline.

#### Use case 2: adding ‘solid’ to cancer queries

In our use case 2, we present an evaluation of a widely adopted approach of reformulating TREC PM queries for clinical trials retrieval by adding terms ‘solid tumor’ with a lower term weight for topics regarding non-blood cancers [32, 33]. We use the expansion weight of 0.2. We evaluate both abstract and clinical trial retrieval tasks. We add the expansion term only to topics which do not contain the word ‘leukemia’ (as leukemia is not a solid tumor cancer). We also skip the expansion for the last 10 topics of the 2019 topic set (as these are diseases other than cancers, so no solid tumors altogether). The pseudocode for the procedure is as follows (a Python implementation and reformulated topics can be found in the Resources page):

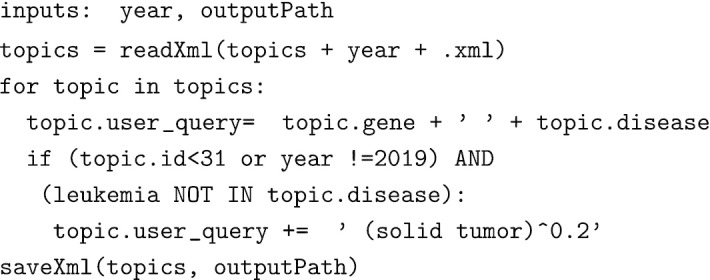



For all evaluations in this use case we use DFR ranking function with parameters $c=1$, $w_{text}=1$ and weights for other index fields set to 0.

The results in Table 5 suggest that ‘solid tumor’ expansion does not work for abstract retrieval, but can be of benefit for clinical trials retrieval, which confirms previous IR research findings [34]. The method’s relative success in clinical trial retrieval can be explained by the fact that some clinical trials explore treatments for a wide variety of cancers, rather than a specific disease. The method brought some improvement for the 2017 and 2018 clinical trials retrieval tasks, but had limited success in the 2019 evaluation.

**Table 5:** Results for use case 2: search effectiveness for runs with and without ‘solid tumor’ expansion on TREC PM collections; run identifiers are *year-task*(*abstracts/*/*clinical trials)-base(w/o expansion)/solid(w expansion)*

Run	RPrec	RR	P@10	InfNDCG
2017-ct-base	0.291	0.566	0.376	–
2017-ct-solid	0.299	0.571	0.393	–
2018-ct-base	0.403	0.728	0.544	0.521
2018-ct-solid	0.411	0.745	0.562	0.530
2019-ct-base	0.432	0.837	0.513	0.584
2019-ct-solid	0.443	0.817	0.510	0.585
2017-abs-base	0.241	0.782	0.467	0.360
2017-abs-solid	0.238	0.749	0.470	0.359
2018-abs-base	0.366	0.785	0.560	0.518
2018-abs-solid	0.363	0.790	0.552	0.515
2019-abs-base	0.323	0.690	0.520	0.491
2019-abs-solid	0.320	0.689	0.520	0.489

**Fig. 4:**
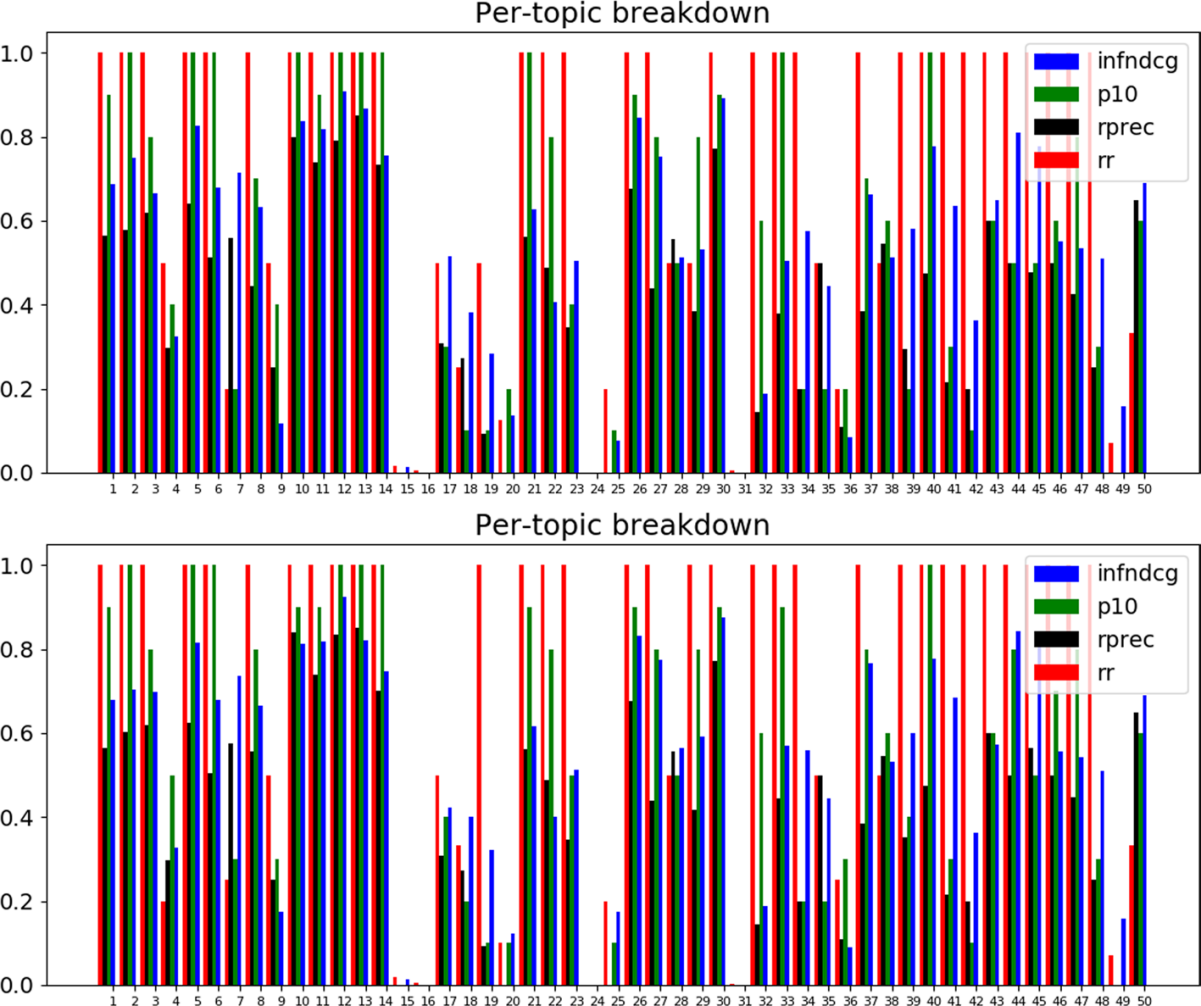
TREC PM 2018 (clinical trials) topic-by-topic results with *solid tumor* expansion (bottom graph) and without *solid tumor* expansion (top graph)

A2A also allows the inspection of the topic-by-topic performance with and without the expansion. An example of such a comparison for TREC PM 2018 (clinical trials) is presented in Fig. 4. The topic-by-topic inspection reveals that the most improvement in P@10 is observed for topic 44 (glioma, BRAF). Glioma, in particular, can be addressed in clinical trials in a number of ways (more general: tumor, brain tumor; more specific: glioblastoma, GBM), so boosting the scores of more general terms (here: ‘tumor’) may prove to be especially helpful.

#### Use case 3: TREC CDS 2015 (task B)

In this use case, we explore using the diagnosis field and objective (test, treatment) to improve abstract retrieval with summaries of medical notes. We evaluate 3 query formulation strategies: (1) summary only, (2) diagnosis and objective and (3) concatenation of all the information—summary, objective and diagnosis. Query formulation strategy for topics with objective ‘diagnosis’ remains unchanged from the default (we use summary only). In all evaluations, we use the same configuration of the search system as in use case 2.

**Table 6:** Results for use case 3: search effectiveness for TREC CDS 2015 Task B (summary) with different query formulation strategies, with queries formulated as different combinations of diagnosis, objective and summary fields

Run	RPrec	RR	P@10	InfNDCG
Summary	0.096	0.562	0.300	0.146
Diagnosis + objective	0.211	0.750	0.427	0.308
Diagnosis + objective + summary	0.112	0.610	0.343	0.173

The results (Table 6) indicate that the diagnosis information improves the retrieval with medical notes. The best results are obtained with the strategy which replaces the note summary with the diagnosis text.

#### Use case 4: Query expansion (QE) using SciSpacy and UMLS

In this use case, we evaluate a QE strategy rooted in using a knowledge base (UMLS) and compare it against a baseline without the expansion and against the automatic query expansion using RM3.

**Table 7:** Results for use case 4: search effectiveness on Genomics 2005 collection; plain BM25 baseline (base) compared to SciSpacy-based NER expansion strategy (NER expansion) and classic PRF (RM3)-based approach

Run	RPrec	RR	P@10
Base	0.214	0.605	0.369
NER expansion	0.136	0.389	0.237
RM3	0.295	0.592	0.447

Query expansion using knowledge bases has been a common theme in biomedical IR systems [33, 35, 36]. In this use case, we evaluate a simple approach in which we use SciSpacy to annotate queries with UMLS concepts. We expand the queries with terms extracted from definitions and synonym sets of all candidate UMLS concepts (linked automatically to named entities detected with SciSpacy).

We run the experiments on the TREC Genomics 2005 dataset. The Python implementation of the query expansion strategy can be found in the Resources page of the A2A application. All the experiments are run with the BM25 ranking function with default parameters ($k_1=1.2$, $b=0.75$) against the *text* index field. For RM3 we use $\alpha =0.3$, $M=20$, $\mu =250$, $k=4$, and we use the *abstract* field to represent the documents when creating the relevance model.

The results in Table 7 indicate that query expansion using UMLS does not work for TREC Genomics 2005 topics. In contrast, RM3 provides a substantial improvement over the non-expanded baseline. Interestingly, the RM3 run performs well enough that it would have been one of the most effective runs of the TREC Genomics 2005 shared task evaluation, had it been submitted. This last finding underlines the importance of being able to experiment with various proven IR models to produce strong and reliable baseline results.

#### Use case 5: free search with RM3

Although A2A is not a live biomedical search engine, it still provides some functionalities that can be of use in advanced literature search. In particular, it allows for the use of pseudo-relevance feedback (RM3) for arbitrary queries.

**Fig. 5:**
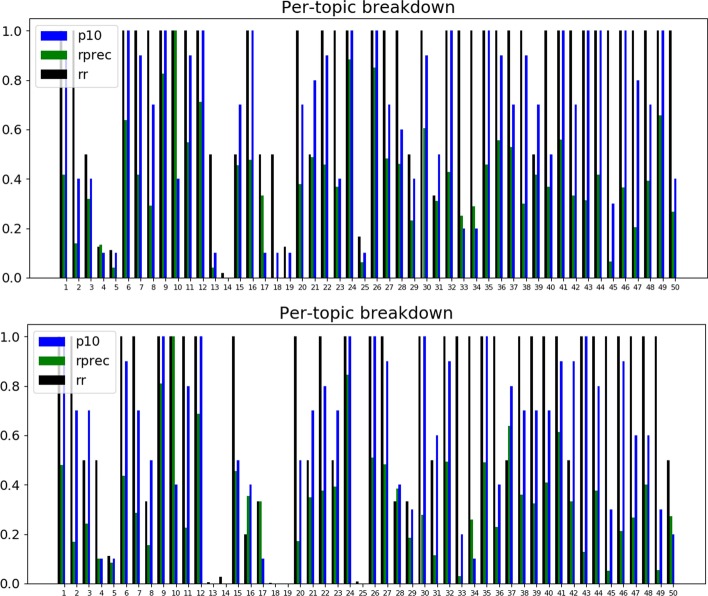
TREC Genomics 2004 topic-by-topic results with RM3 expansion (bottom graph) and without RM3 expansion (top graph)

This can be motivated with a specific example. If we consider topic 49 (query about ‘glyphosate tolerance gene sequence’) of TREC Genomics 2004, we can see that, with RM3, the number of relevant documents retrieved at 10 rises to 10 from 3 out of 10 (see Fig. 5 for topic-by-topic comparison; here we compare the blue bar, P@10, for topic 49 across top and bottom graphs).

**Fig. 6:**
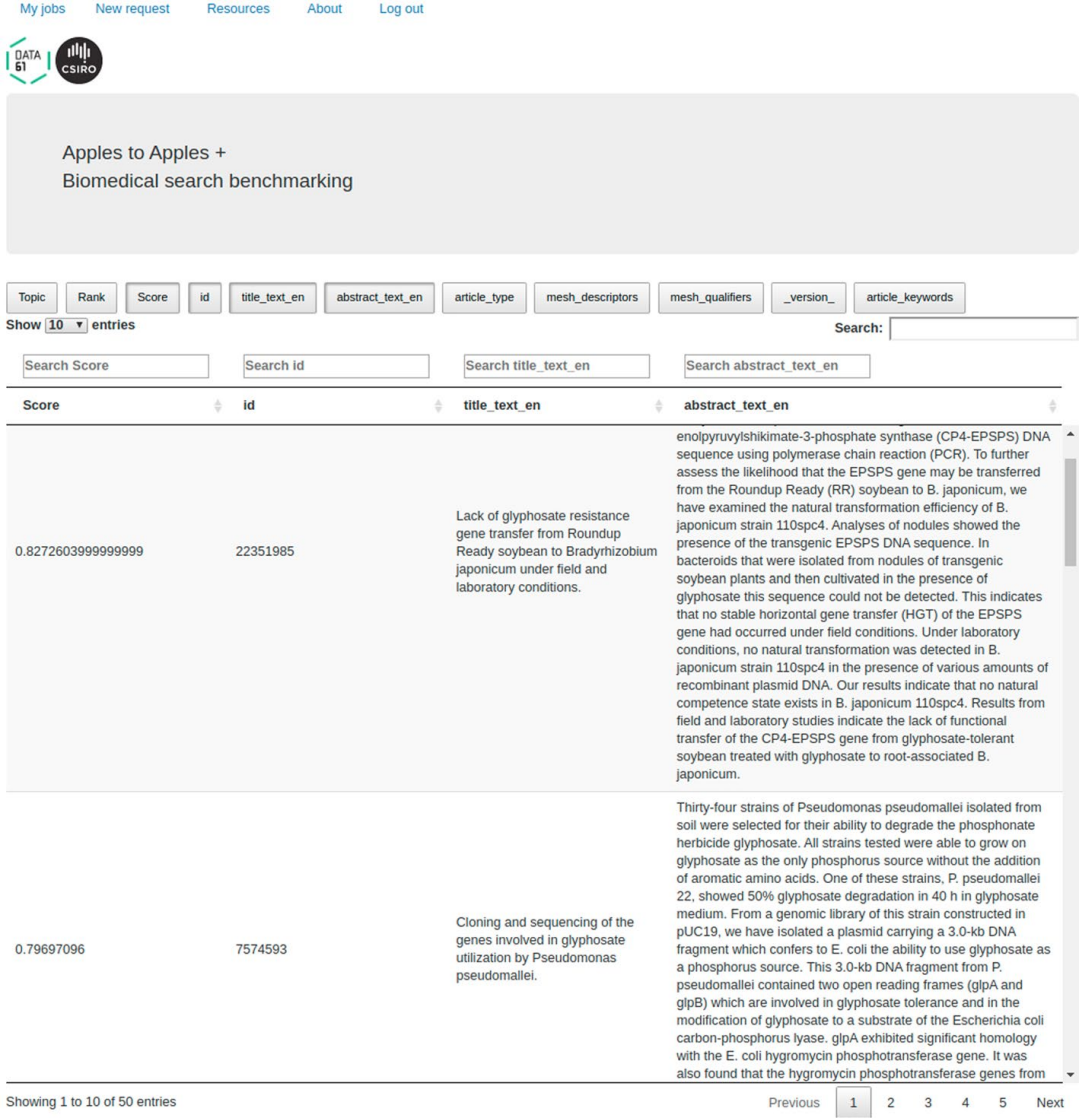
Results preview for Use Case 5—a document view

Let’s consider a user who wants to seek answers to this particular query using RM3 against a newer (than 2004) document collection. The hypothetical user can encode it as a topic file with a user_query field (see *2004_small.xml* file in the Resources page) and evaluate it against any of the indexed collections using RM3 query expansion. It can be noted that any other query can be submitted to the system, so the topic 49 from TREC Genomics 2004 merely serves as an illustrative example. Since the query will be evaluated against ‘wrong’ relevance judgements (we do not have human judgements for this topic for any collection other than TREC Genomics 2004), the evaluation results will be meaningless, but A2A provides a download link for full TREC results (1000 documents per topic) and an interface to explore the top results. In Table 8, we present the IDs of the top 5 documents retrieved for the query from topic 49 (2004) with RM3 from the 2019’s PubMed corpus (titles and years were appended manually; topical relevance was assessed upon reading the corresponding abstract). Figure 6 presents the corresponding application screen (results view; results 2 and 3 from the table are shown with corresponding abstracts). Since this methodology can be generalised for any information need, A2A provides access to otherwise unavailable biomedical IR methods (such as RM3) for any correctly formatted topic file.

**Table 8:** Results for use case 5: documents retrieved by searching with a Genomics 2004 topic over 2019 PubMed collection using RM3 for query expansion

ID	Title	Year	Relevance
25474478	Isolation, Cloning, and Characterization of a Partial Novel Aro A Gene in Common Reed (Phragmites Australis)	2015	$+$
22351985	Lack of Glyphosate Resistance Gene Transfer From Roundup Ready Soybean to Bradyrhizobium Japonicum Under Field and Laboratory Conditions	2011	$+$
7574593	Cloning and Sequencing of the Genes Involved in Glyphosate Utilization by Pseudomonas Pseudomallei	1995	$+$
24836188	Functional Characterization of aroA From Rhizobium Leguminosarum With Significant Glyphosate Tolerance in Transgenic Arabidopsis	2014	$+$
26411727	Multiple Mechanism Confers Natural Tolerance of Three Lilyturf Species to Glyphosate	2016	$+$

### Advantages and limitations

The main advantage of A2A is that it allows running a wide range of information retrieval experiments within the life sciences domain, without the need for cumbersome local deployment of a complicated and resource-intensive system. To provide some perspective, the Solr server that A2A uses employs over 135 GB of disk space. The indexing involves handling raw data files, which requires a lot of additional free disk space for the indexing process. To make things more complex, a running Solr server can operate only on a single set of parameters for a specific collection in any given moment. This means that a deployment effort for a shared server (e.g., intranet deployment) would have to involve implementing a queue to handle search requests without conflicts (e.g., user A switching configuration during an ongoing task execution of user B). Furthermore, a software wrapper is needed for the server to change search parameters. Moreover, the indexing itself is also time-consuming, as each collection comes in a different format and requires different parsers.

All this turns biomedical information retrieval research into a troublesome mix of software engineering, data wrangling and software deployment. At the same time, some parts of information retrieval research—such as query reformulation—require more than a good idea and a script to transform topic texts into queries. For end-users without any programming or software development expertise, A2A provides a tool to benchmark their search strategies (for example, via manual query reformulation with rules similar to the one in use case 2).

For end-users who wish to experiment with new methods for automatic query expansion, A2A bridges the gap between (either basic or not-so-basic) text engineering and IR research (e.g., the entity linking idea from use case 4).

For end-users who have access to local infrastructure for running IR experiments, A2A can serve as a time-saving tool for obtaining reproducible (and bug-free) baseline scores for their experiments—such as the RM3 baseline from use case 4. This way, our system reduces the need for re-implementing (or re-factoring) well known (but hardly available as existing implementations) methods such as RM3, or demographic matching for clinical trials.

For all user profiles, A2A offers a simple to understand presentation of evaluation results using some of the most popular evaluation metrics. In particular, all topic-by-topic search effectiveness graphs presented in this paper were plotted by the A2A web application.

Finally, A2A provides a fully parameterisable search system over a wide range of biomedical corpora. Although our core focus is on benchmarking and not search, we believe that A2A can be used in some literature search scenarios (see use case 5).

It is also worth noting that, by design, with user-submitted topics (either reformulated or ‘free’ search queries), A2A supports the full query syntax of edismax query parser. This means that queries can be reformulated with AND/OR operators, mandatory and optional terms, boosts defined per index field-term pairs, negative boosting, term negation (i.e., matching for documents which do not contain a given term), distance-based queries, phrase queries, wildcards and many other features of the Solr query syntax.

As for the limitations of the system, a few are inherent to the design of A2A as a benchmarking tool, while most can be labeled as ‘future and ongoing work’. We first describe the true limitations.

By definition, A2A operates on ‘closed’ test collections, which means that we do not perform live indexing of new documents, thus putting a ceiling to the usability of A2A as a fully-fledged search system.

Another limitation is the adaptability of the tool to various IR research scenarios. We delegate some of the key research problems to offline processing (query expansion now, document re-ranking in future) to make the tool adaptable to the needs of a wide community interested in biomedical IR research. Nonetheless, there are limits to this adaptability—using A2A means using the underlying indexes and search engine. There is no way to experiment, for example, with some of the design choices that produce an effect at indexing time.

We now turn to those limitations that we intend to address as future work. Although we do not aim to change A2A into a live search system, a basic browsing interface to explore the search results for individual topics at a document level is already included in the platform. We plan to extend it to support browsing through large quantities of documents (i.e., the full results, rather than only the top 50 documents per topic). Similarly, adding the possibility of uploading TREC-formatted result files for direct evaluation will mark an important step towards facilitating research in results re-ranking. In such cases A2A would allow for offline manipulation on a previously downloaded results file (according to a given logic), before submitting it for re-evaluation (i.e., evaluation of re-ranking). It is another functionality we expect to include shortly in the system, possibly with some predefined re-ranking and rank-fusion models.

Other future work includes incorporating the most recent biomedically-themed TREC evaluations, such as TREC-COVID or TREC PM 2020.

### Comparison with other related systems

A2A has several features that set it apart from other systems designed to improve reproducibility in IR research, such as Anserini [21]. They are: (1) a web-based GUI; (2) no need for local deployment; and, (3) the in-built support for biomedical IR benchmarking collections. Similar to Anserini, A2A benefits from using a well established search engine at its core. Both systems are based on Lucene—directly, as in the case of Anserini, or indirectly through Solr in the case of A2A.

A2A can also be compared, although to a lesser extent, to broad purpose biomedical search tools such as PubMed. For users looking for a literature search and browsing functionality on an up-to-date collection of documents, in most cases PubMed is a better choice. A2A provides a basic search interface (i.e., a screen for browsing the results), which means that a user can potentially seek an answer to a given information need. Nonetheless, our system does not operate on a live collection of documents, but on benchmark collections instead. The advantage of A2A, therefore, lies in its capacity of measuring search effectiveness against human judgement. This is useful in search benchmarking rather than in actual retrieval.

## Conclusions

We provide a software platform, called A2A, which allows researchers of various backgrounds to benchmark biomedical IR systems. In order to perform a comprehensive assessment of different search methodologies, A2A allows the user to formally specify the methods they use, re-run past experiments, and analyse the findings based on the official TREC evaluation framework. The methods include: BM25 and DFR ranking functions, RM3 query expansion, and demographic matching. A2A allows users to customise the query formulation process. Its graphical user interface allows for detailed qualitative and quantitative analysis of the retrieval performance.

By using our platform, IR researchers will be able to both report their methods in a consistent way, and evaluate their results against a common baseline. In the future, we aim to use this platform to systematically evaluate a more diverse combination of search methods.

## Data Availability

All the datasets used in this work are available through NIST TREC website: https://trec.nist.gov/data.html. Additionally, all the scripts used for topic processing with example outputs prominent in presented use cases are made available (publicly; to registered users) at: https://a2a.csiro.au/benchmarking/resources. The A2A tool is available at https://a2a.csiro.au/benchmarking/about. The system requires registration.

## Abbreviations

TREC: Text REtrieval Conference
CDC: Clinical Decision Support
NIST: National Institute of Standards and Technology
A2A: Apples to Apples
IR: Information Retrieval
PRF: Pseudo-Relevance Feedback
RM: Relevance Model
PM: Precision Medicine
UMLS: Unified Medical Language System
NER: Named Entity Recognition
GUI: Graphical User Interface

## Footnotes

1 We use ‘biomedical’ as an umbrella term, covering both ‘bio’ and ‘medical’ and the in-between.

2 https://a2a.csiro.au/benchmarking/about.

3 EvaluatIR at www.evaluatIR.org.

4 https://lucene.apache.org/solr/ (accessed 30 Oct 2020).

5 https://lucene.apache.org/ (accessed 30 Oct 2020).

6 https://www.elastic.co/ (accessed 30 Oct 2020).

7 SIGIR is the Association for Computing Machinery’s Special Interest Group on Information Retrieval: https://sigir.org/ (accessed 7 Sep 2020).

8 https://metamap.nlm.nih.gov/MetaMapLite.shtml (accessed 7 Sep 2020).

9 Bag is an extension of a set, which allows for presence of multiple equal elements.

10 https://lucene.apache.org/solr/guide/8_2/the-extended-dismax-query-parser.html.

11 https://datatables.net/ and allows for a fully customised display of the document ranking (including column order and visibility, sorting and filtering rows, and pagination control).

## Availability and requirements

Project name: A2A-search benchmarking for life sciences

Project home page: https://a2a.csiro.au/benchmarking/about

Operating system(s): Platform independent (client), Linux (server)

Programming language: Python 3.8

Other requirements: A web browser, preferably Google Chrome; one query reformulations scripts (downloadable from Resources page of the tool) requires installation of SciSpacy dependencies; all scripts were tested on Ubuntu 18.10

License: CC

Any restrictions to use by non-academics: None
